# Characteristic metabolites of *Hypericum* plants: their chemical structures and biological activities

**DOI:** 10.1007/s11418-021-01489-y

**Published:** 2021-02-08

**Authors:** Naonobu Tanaka, Yoshiki Kashiwada

**Affiliations:** Graduate School of Pharmaceutical Sciences, Tokushima University, Tokushima, 770-8505 Japan

**Keywords:** *Hypericum*, Hypericaceae, Characteristic metabolite, Chemical structure, Biological activity

## Abstract

Plants belonging to the genus *Hypericum* (Hypericaceae) are recognized as an abundant source of natural products with interesting chemical structures and intriguing biological activities. In the course of our continuing study on constituents of *Hypericum* plants, aiming at searching natural product-based lead compounds for therapeutic agents, we have isolated more than 100 new characteristic metabolites classified as prenylated acylphloroglucinols, meroterpenes, ketides, dibenzo-1,4-dioxane derivatives, and xanthones including prenylated xanthones, phenylxanthones, and xanthonolignoids from 11 *Hypericum* plants and one *Triadenum* plant collected in Japan, China, and Uzbekistan or cultivated in Japan. This review summarizes their chemical structures and biological activities.

## Introduction

*Hypericum* plants of the family Hypericaceae, consisting of over 500 perennial herbs or shrubs subdivided into 30 sections, are mainly distributed in temperate area [1]. Some of *Hypericum* plants have been used as traditional remedies in various parts of the world. A number of researches on the constituents of *Hypericum* plants have resulted in the isolation of various classes of natural products including terpenoids, flavonoids, xanthones, naphthodianthrones, and prenylated acylphloroglucinols (PAPs) [2]. Among others, hypericin, a naphthodianthrone derivative found in *Hypericum* plants belonging to the sections *Hypericum*, *Adenotras*, and *Drosocarpium*, is recognized as one of the most potent naturally occurring photodynamic agents [3]. PAPs are specialized metabolites of plants belonging to some genera of the Hypericaceae and Clusiaceae families including *Hypericum*, *Garcinia*, *Clusia*, and so on [4–6], while several meroterpenes structurally and biosynthetically related to PAPs have also been reported from these plant species [7]. Since diverse and complex chemical structures and intriguing biological activities of the PAPs have attracted huge interests of researchers, some excellent systematic reviews for PAPs have been published [4–6, 8].

Our research group has been conducting a study searching for new plant metabolites with unique chemical structures and biological activities [9–11]. In the course of this research project, we investigated 11 *Hypericum* species belonging to the sections *Roscyna* (*H. ascyron*), *Ascyreia* (*H. monogynum* and *H. patulum*), *Hypericum* (*H. sikokumontanum*, *H. kiusianum*, *H. yojiroanum*, *H. yezoense*, and *H. erectum*), *Myriandra* (*H. frondosum* ‘Sunburst’), *Elodeoida* (*H. elodeoides*), and *Hirtella* (*H. scabrum*) collected in Japan, China, and Uzbekistan or cultivated in Japan together with one species of *Triadenum* (*T. japonicum*), a sister genus of *Hypericum*, to isolate more than 100 of new characteristic metabolites. In this review, their chemical structures and biological activities as well as related studies conducted by other research groups are summarized.

## PAPs, prenylated xanthones, and dibenzo-1,4-dioxane from *Hypericum ascyron* (section *Roscyna*)

**Fig. 1:**
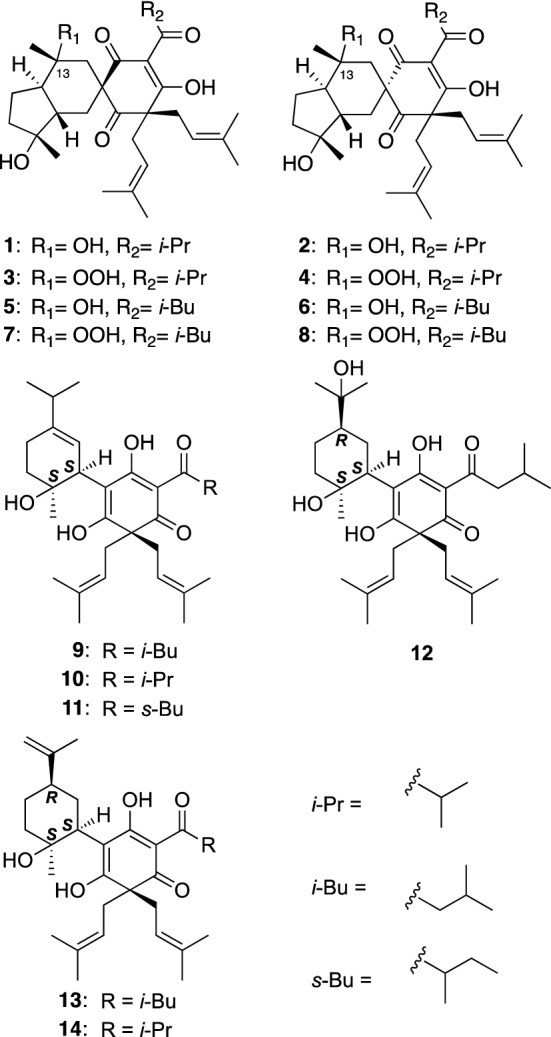
The structures of tomoeones A–H (**1**–**8**), hypascyrins A–E (**9**–**13**), and *ent*-hyphenrone J (**14**) isolated from *Hypericum ascyron*

*Hypericum ascyron* (Tomoesou in Japanese) is a perennial herb widely distributed in eastern Asia, and the whole plants have been used as an herbal medicine to treat headache, wounds, and abscesses in China. The whole plants of *H. ascyron* collected in Tokushima prefecture, Japan were separated into the aerial parts and roots. Their chemical constituents were separately investigated by chromatographic techniques to isolate some PAPs (**1**–**15**). Their structures were established based on spectroscopic analyses. Tomoeones A–H (**1**–**8**) isolated from the aerial parts of *H. ascyron* were assigned as the first example of spirocyclic PAPs (Fig. 1) [12], whereas about 50 related spirocyclic PAPs have been isolated from some *Hypericum* plants to date [4]. The hydroxy substituents and the relative configurations of C-13 in tomoeones C (**3**), D (**4**), G (**7**), and H (**8**) have been revised by Zhang et al. [13]. Antiproliferative activity of tomoeones A–H (**1**–**8**) against human tumor cell lines including multidrug-resistant (MDR) cancer cell lines was evaluated to show a significant cytotoxicity of **6** against KB cells with an IC_50_ value of 6.2 μM [12]. Tomoeone F (**6**) also exhibited antiproliferative activity against MDR cancer cell lines (KB-C2 and K562/Adr), which was more potent than doxorubicin.

Investigation of *H. ascyron* roots gave six new PAPs with menthane moieties, hypascyrins A–E (**9**–**13**) and *ent*-hyphenrone J (**14**) (Fig. 1) [14]. The absolute configuration of **9** was deduced by comparison of experimental and time-dependent density functional theory (TDDFT) calculated electronic circular dichroism (ECD) spectra, while those of **10**–**14** were assigned by ECD analyses as well as chemical conversions. Hypascyrins A (**9**), C (**11**), and E (**13**), and *ent*-hyphenrone J (**14**) exhibited potent antimicrobial activities against methicillin-resistant *Staphylococcus aureus* (MRSA) (MIC_50_ values of 4.0, 8.0, 2.0, and 4.0 μM, respectively, for seven strains) and *Bacillus subtilis* (MIC values of 4.0, 4.0, 2.0, and 4.0 μM, respectively).

**Fig. 2:**
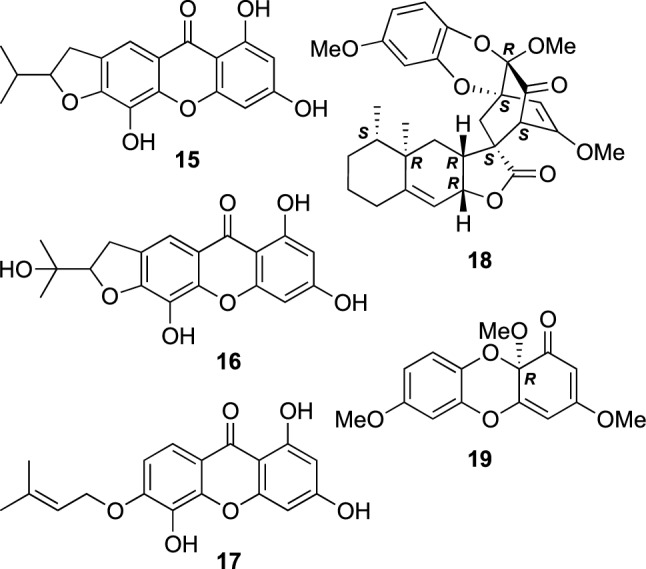
The structures of prenylated xanthones (**15**–**17**) and hyperdioxanes A (**18**) and B (**19**) isolated from *Hypericum ascyron*

*Hypericum* plants are known to be a rich source of aromatic compounds including xanthones. Some prenylated xanthones, 1,3,5-trihydroxy-6,7-[2′-(1-methylethenyl)-dihydrofurano]-xanthone (**15**), 1,3,5-trihydroxy-6,7-[2′-(1-hydroxy-1-methylethyl)-dihydrofurano]-xanthone (**16**), and 1,3,5-trihydroxy-6-*O*-prenylxanthone (**17**) were isolated from the aerial parts of *H. ascyron* (Fig. 2) [15]. In contrast, the roots of *H. ascyron* were studied to isolate two naturally rare dibenzo-1,4-dioxane derivatives, hyperdioxanes A (**18**) and B (**19**) (Fig. 2) [16]. Hyperdioxane A (**18**) is a unique conjugate of **19** and a sesquiterpene, eremophil-9,11(13)-dien-8β,12-olide, possessing an unprecedented heptacyclic ring system. The structures of **18** and **19** were assigned by detailed spectroscopic analyses, including application of a modified Mosher’s method to a derivative of **19**. An evaluation of biological activity of **18** and **19** is ongoing.

## PAPs, meroterpenes, and xanthones from *Hypericum monogynum *and *H. patulum* (section *Ascyreia*)

**Fig. 3:**
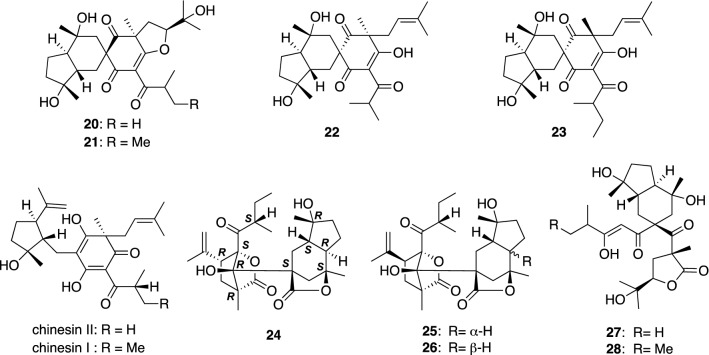
The structures of chipericumins A–D (**20**–**23**) and biyoulactones A–E (**24**–**28**) as well as chinesins I and II isolated from *Hypericum monogynum*

*Hypericum monogynum* (syn. *H. chinense* var. *salicifolium*) (Biyouyanagi in Japanese), an evergreen shrub originated in China, is cultivated as an ornamental plant in Japan. Its stems and leaves have been used for the treatment of female disorders in Japan. In contrast, the roots of this plant have been used to treat various disorders, such as rheumatism, snakebite, and furuncle, in China. Chemical constituents of the roots, stems, and leaves of *H. monogynum* cultivated in Tokushima prefecture were separately and thoroughly investigated to isolate new characteristic metabolites. Chipericumins A–D (**20**–**23**) are spirocyclic PAPs isolated from the roots (Fig. 3) [17], of which chipericumins A (**20**) and B (**21**) have a unique 5/6/6/5 tetracyclic ring system. Chinesins I and II (Fig. 3), PAPs previously isolated from the same plant by Tada et al. [18], might be biogenetic precursors of **20**–**23**. Unique meroterpenes structurally related to **20**–**23**, biyoulactones A–E (**24**–**28**), were also isolated from the roots of *H. monogynum*. Among others, biyoulactones A–C (**24**–**26**) are novel pentacyclic meroterpenes possessing bi- and tricyclic γ-lactone moieties connected through a C–C single bond [19]. The structure including the absolute configuration of biyoulactone A (**24**) was assigned by a combination of NMR and single crystal X-ray diffraction analyses. Biyoulactones D (**27**) and E (**28**) are PAP-related meroterpenes having an octahydroindene ring, a γ-butyrolactone ring, and an enolized β-diketone moiety [20]. Their relative configurations were deduced based on NOESY data aided with computational conformational analysis.

**Fig. 4:**
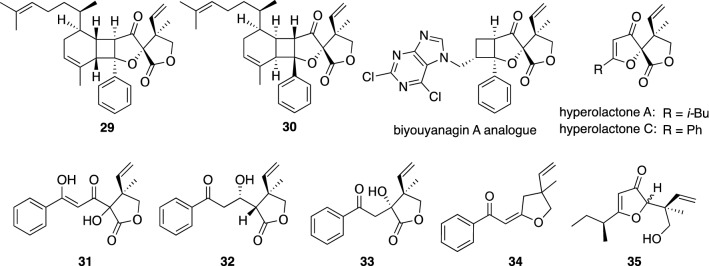
The structures of biyouyanagins A (**29**) and B (**30**), 5,6-dihydrohyperolactone D (**31**), 4-hydroxyhyperolactone D (**32**), and merohyperins A–C (**33**–**35**) isolated from *Hypericum monogynum* as well as biyouyanagin A analogue and hyperolactones A and C

From the leaves of *H. monogynum*, we isolated biyouyanagins A (**29**) and B (**30**) (Fig. 4) [21, 22], novel meroterpenes possessing a unique 6/4/5/5 tetracyclic ring system including a spiro-lactone moiety, and proposed their biogenetic pathway from a sesquiterpene (*ent*-zingiberene) and a spiro-lactone derivative (hyperolactone C), of which the latter had been reported from the same plant by Tada et al. [23] (Fig. 4). The total syntheses of **29** and **30** proceeded by Nicolaou et al. resulted in the revision of the stereochemistries of **29** and **30** [24–26]. Xie et al. also achieved the total synthesis of **29** [27]. Biyouyanagin A (**29**) exhibited a potent and selective inhibitory effect on HIV replication in H9 lymphocytes with therapeutic index (TI) value of > 31.3 [21]. Furthermore, **29** inhibited LPS-induced cytokine productions (IL-10, IL-12, and TNF-α) from peripheral blood mononuclear cells [21]. An analogue of biyouyanagin A (**29**) possessing more potent biological activity was discovered by Nicolaou et al. in their synthetic study on analogues of **29** [28, 29]. 5,6-Dihydrohyperolactone D (**31**) and 4-hydroxyhyperolactone D (**32**) are simple linear meroterpenes co-isolated with biyouyanagins (Fig. 4) [22], while Xie et al. reported the biomimetic synthesis of **32** [30].

Further investigation on the constituents of *H. monogynum* leaves gave merohyperins A–C (**33**–**35**) (Fig. 4) [31], of which merohyperins A (**33**) and B (**34**) had a novel carbon skeleton. Comparison of the experimental and DFT calculated ^13^C NMR data implied the geometory of a double bound in **34** to be *E*. Merohyperin C (**35**) was obtained as a separable epimeric mixture, and the structure of **35** was assigned by chemical conversion of a known meroterpene, hyperolactone A (Fig. 4) [23] into **35** [31].

**Fig. 5:**
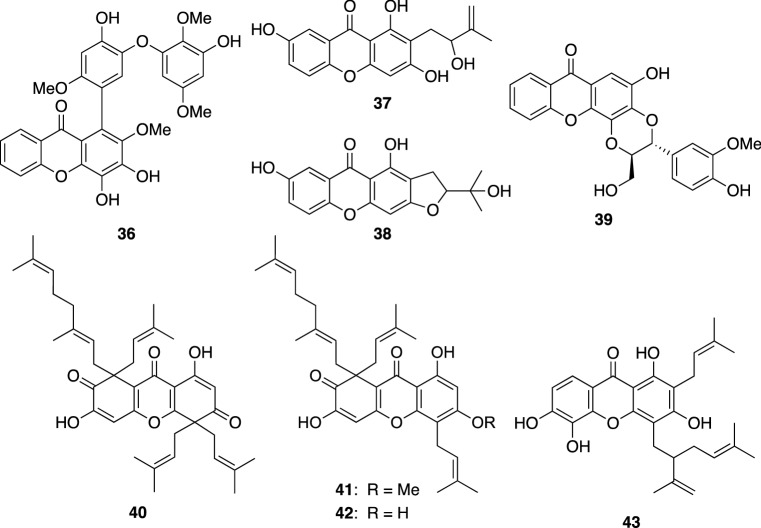
The structures of chinexanthone (**36**), prenylated xanthones (**37** and **38**), 2-demethylkielcorin (**39**), and biyouxanthones A–D (**40**–**43**) isolated from *Hypericum monogynum*

We reported the isolation of about 50 xanthones from the leaves and stems of *H. monogyum* [32–35], of which one was phenylxanthone, four were prenylated xanthones, five were xanthonolignoids, and others were simple xanthones with hydroxy and/or methoxy groups. Among them, chinexanthone (**36**), two prenylated xanthones (**37** and **38**), and 2-demethylkielcorin (**39**) were new compounds (Fig. 5). Ten simple xanthones, 4,6-dihydroxy-2,3-dimethoxyxanthone, 2,6-dihydroxy-3,4-dimethoxyxanthone, 6-hydroxy-2,3,4-trimethoxyxanthone, 3,6-dihydroxy-1,2-dimethoxyxanthone, 4,7-dihydroxy-2,3-dimethoxyxanthone, 3,7-dihydroxy-2,4-dimethoxyxanthone, 1,3,7-trihydroxy-5-methoxyxanthone, 1,7-dihydroxy-5,6-dimethoxyxanthone, 4,5-dihydroxy-2,3-dimethoxyxanthone, and 1,3-dihydroxy-2,4-dimethoxyxanthone, were also identified to be new compounds [32, 33]. Chinexanthone (**36**), possessing a phenyl substituent in xanthone skeleton, appeared to be a new class of xanthones as phenylxanthone [34]. Many xanthonolignoid, a class of xanthone fused with a C_6_-C_3_ moiety forming a 1,4-dioxane ring, reported previously were isolated as racemic mixtures. In contrast, the xanthonolignoids including 2-*O*-demethylkielcorin (**39**) isolated by our study were shown to be a partial racemate {[α]_D_ + 15.4 (*c* 0.5, MeOH)}. Assignments of the absolute configuration for the major enantiomer of **39** as well as the ratio of enantiomers (88:12) were elucidated by analyzing their MTPA ester derivatives [34]. We evaluated antiproliferative activities of the xanthones isolated from *H. monogynum* against a panel of human cancer cell lines including MDR human cancer cell lines [34]. Though most xanthones were non-cytotoxic, some xanthones were shown to be more toxic against MDR cancer cells.

Biyouxanthones A–D (**40**–**43**) are highly prenylated xanthones isolated from the roots of *H. monogynum* (Fig. 5) [35]*.* Biyouxanthones A (**40**) and B (**41**) inhibited the hepatitis C virus (HCV) core protein level in the culture of HCV-infected human hepatoma Huh7 cells (89% and 61%, respectively) at 10 μM. Luo et al. showed a neuroprotective effect against corticosterone-induced lesions of PC12 cells and an inhibitory effect on NO production in LPS-induced BV2 microglia cells of biyouxanthone D (**43**) [36].

**Fig. 6:**
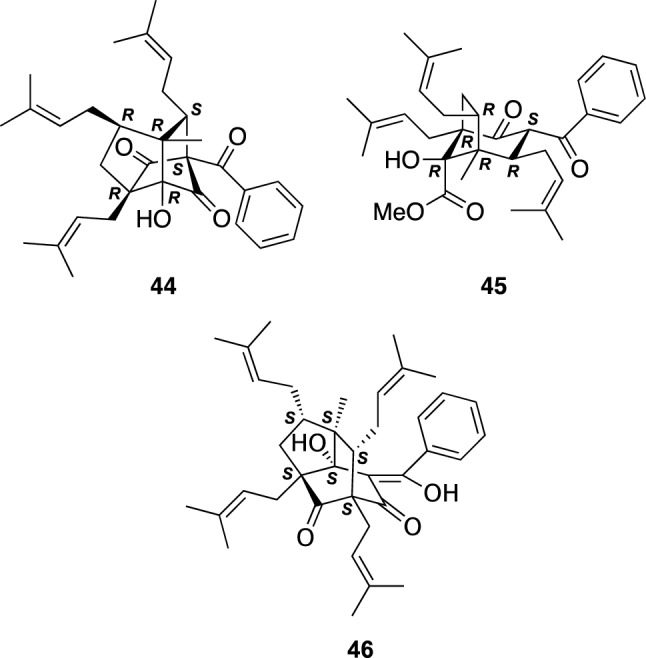
The structures of hypatulins A–C (**44**–**46**) isolated from *Hypericum patulum*

Two PAP-related meroterpenes, hypatulins A (**44**) and B (**45**), and a PAP, hypatulin C (**46**), were isolated from the leaves of *H. patulum* (Kinshibai in Japanese), an evergreen shrub originated from China (Fig. 6) [37, 38]. Hypatulin A (**44**) had a unique densely substituted tricyclic octahydro-1,5-methanopentalene core. The absolute configuration of **44** was elucidated on the basis of TDDFT calculation of ECD spectrum, while chemical conversion of **44** into **45** led to the assignment of that of **45**. Hypatulin C (**46**) had a tricyclic [4.3.1.0^3,7^]-decane core highly substituted by prenyl groups, whose absolute configuration was also deduced on the basis of ECD calculation. Hypatulin A (**44**) exhibited a moderate antimicrobial activity against *B. subtilis* [37].

## PAPs, chromone glucosides, chromanone glucosides, and meroterpenes from *Hypericum sikokumontanum*, *H*.* kiusianum*, *H. yojiroanum*, *H. yezoense*, and *H. erectum *(section *Hypericum*)

**Fig. 7:**
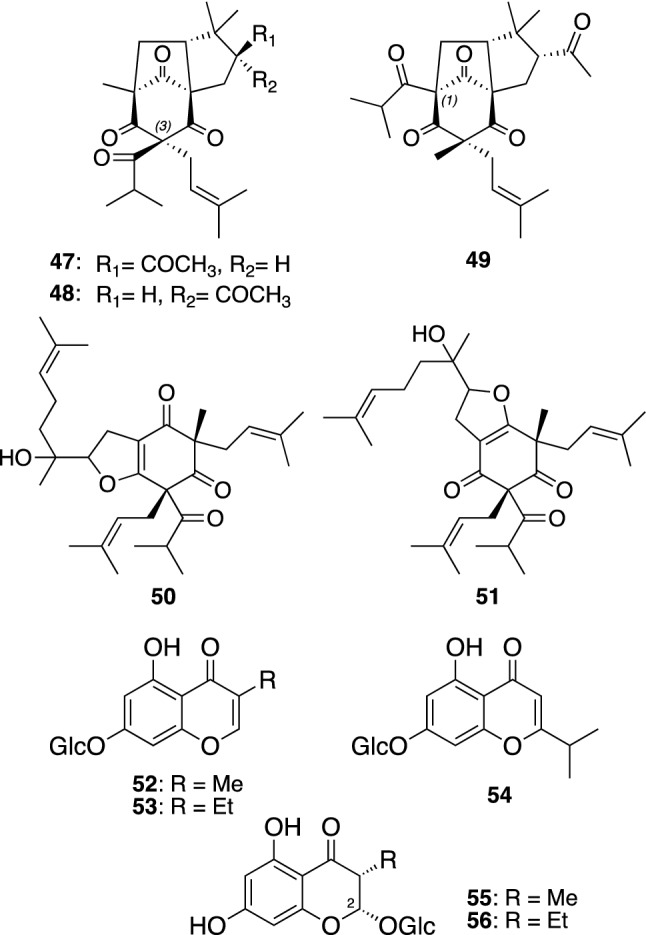
The structures of takaneones A–C (**47**–**49**), takaneols A (**50**) and B (**51**), takanechromones A–C (**52**–**54**), and takanechromanones A (**55**) and B (**56**) isolated from *Hypericum sikokumontanum*

*Hypericum sikokumontanum* (Takane-otogiri in Japanese) is a perennial herb grown on mountain areas more than 1,400 m above the sea level in Shikoku island, Japan. Phytochemical investigation of the aerial parts of *H. sikokumontanum* afforded five PAPs, three chromone glucosides, and two chromanone glucosides [39, 40]. Takaneones A–C (**47**–**49**) are PAPs possessing a tricyclic moiety including a bicyclo[3.2.1]octane-2,4,8-trione core with a characteristic C_4_ alkyl moiety (Fig. 7) [39]. Although a large number of polycyclic PAPs possessing a bicyclo[3.3.1]nonane-2,4,9-trione or bicyclo[3.2.1]octane-2,4,8-trione have been reported from various Hypericaceous and Clusiaceous plants, they could be divided into two classes (types A and B) depending on the relative position of the acyl group on the phloroglucinol moiety [4, 5]. Namely type A PAPs have the acyl groups at C-1 position of their phloroglucinol moieties, while the acyl groups of type B PAPs are located at C-3 position [4]. Takaneones A (**47**) and B (**48**) are type B PAPs, whereas takaneone C (**49**) is the first example of type A PAP with a bicyclo[3.2.1]octane-2,4,8-trione core. Takaneols A (**50**) and B (**51**) are PAPs with a dihydrofuran moiety fused to the phloroglucinol moiety [39]. The enantiospecific synthesis of the tricyclic core of takaneones A–C (**47**–**49**) was conducted by Srikrishna et al. [41]. Takaneones B (**48**) and C (**49**) and takaneol A (**50**) showed cytotoxicities against K562/Adr MDR cancer cells with IC_50_ values ranging from 4.7 to 10.0 μg/mL, which were slightly more potent than doxorubicin. Their potency of cytotoxicity against MDR cancer cell lines (KB-C2 and K562/Adr) was similar to those against sensitive cell lines (KB and K562) [39].

Takanechromones A–C (**52**–**54**) and takanechromanones A (**55**) and B (**56**) are simple chromone glucosides and chromanone glucosides, respectively (Fig. 7) [40]. They are considered to be cyclized products of acylphloroglucinols with amino acid-derived acyl starters, and **55** and **56** are the first 2-hydroxychromanone derivatives from natural source [42]. 5,7-Dihydroxy-3-methylchromone and 5,7-dihydroxy-3-ethylchromone, aglycones of **52** and **53**, respectively, co-isolated with **52**–**56** in our study, exhibited an antimicrobial activity against *Helicobacter pylori* and antiproliferative activities against MDR cancer cell lines [40]. Takanechromone C (**54**) was also isolated from a Rosaceous plant *Agrimonia pilosa* by Li et al*.* [43].

**Fig. 8:**
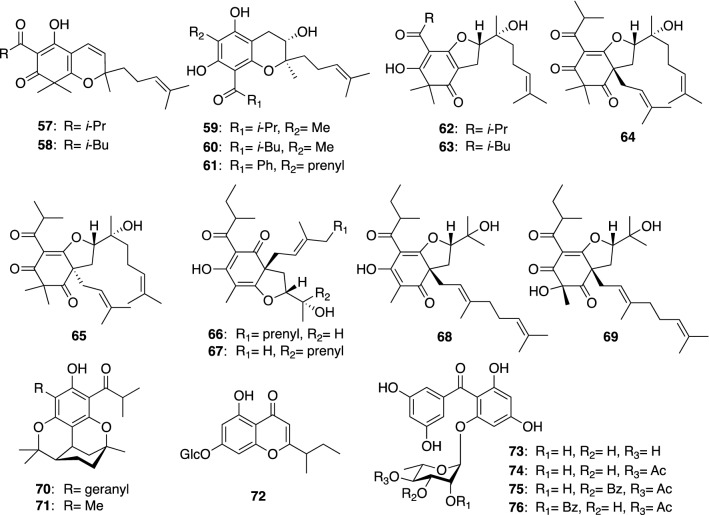
The structures of petiolins A–C (**57**, **58**, and **62**), D (**70**), E (**72**), F–I (**73**–**76**), J (**59**), L (**64**), and M (**65**) isolated from *Hypericum kiusianum* and yojironins C (**63**), D (**60**), E–H (**66**–**69**), and I (**61**) isolated from *H. yojiroanum*

**Fig. 9:**
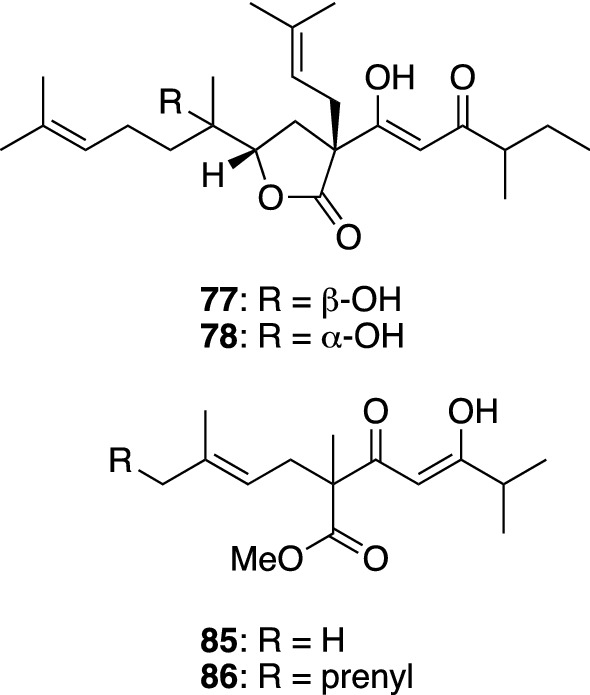
The structures of yojironins A (**77**) and B (**78**) isolated from *Hypericum yojiroanum* and yezo’otogirins G (**85**) and H (**86**) isolated from *H. yezoense*

*Hypericum kiusianum* (syn. *H. pseudopetiolatum* var. *kiusianum*) (Nagasaki-otogiri in Japanese) is a perennial herb distributed mainly in Kyushu and Shikoku islands, Japan, while a small perennial herb *H. yojiroanum* (Daisetsuhina-otogiri in Japanese) grows in Hokkaido, Japan. From the aerial parts of *H. kiusianum* collected at Kochi prefecture and the purchased whole plants of *H. yojiroanum*, we isolated a series of simple bicyclic PAPs named petiolins A–C (**57**, **58**, and **62**), J (**59**), L (**64**), and M (**65**) and yojironins C (**63**), D (**60**), E (**66**), F (**67**), G (**68**), H (**69**), and I (**61**) (Fig. 8) [44–47]. Petiolins A–C (**57**, **58**, and **62**) showed a moderate cytotoxicity against human epidermoid carcinoma KB cells [44]. Petiolin C (**62**) also exhibited a weak antifungal activity against *Trichophyton mentagrophytes*, whereas petiolin J (**59**) showed antimicrobial activities against *Micrococcus luteus*, *Cryptococcus neoformans*, and *T. mentagrophytes* [45]. Petiolins D (**70**) and K (**71**) are racemic tetracyclic PAPs with the citran skeleton isolated from *H. kiusianum*, whose structures were elucidated by X-ray crystallographic analyses [45, 48]. In addition to the PAPs mentioned above, a chromone glucoside, petiolin E (**72**), and benzophenone rhamnosides, petiolins F–I (**73**–**76**), were isolated from *H. kiusianum* [48, 49]. Recently, Wang et al*.* isolated petiolin G (**74**) from another *Hypericum* plant (*H. wightianum*) and reported its neuroprotective effect against corticosterone-induced PC12 cell injury [50]. Yojironins A (**77**) and B (**78**), isolated from *H. yojiroanum*, are biogenetically unique meroterpenes (Fig. 9) [46], being composed of only two acetate units with a 2-methylbutanoyl group and three isoprene units. Yojironin A (**77**) exhibited potent antimicrobial activities against *Aspergillus niger* (IC_50_ 8 μg/mL), *Candida albicans* (IC_50_ 2 μg/mL), *Cryptococcus neoformans* (IC_50_ 4 μg/mL), *T. mentagrophytes* (IC_50_ 2 μg/mL), *S. aureus* (MIC 8 μg/mL), and *Bacillus subtilis* (MIC 4 μg/mL) as well as antiproliferative activities against KB cells and murine lymphoma L1210 cells in vitro [46].

**Fig. 10:**
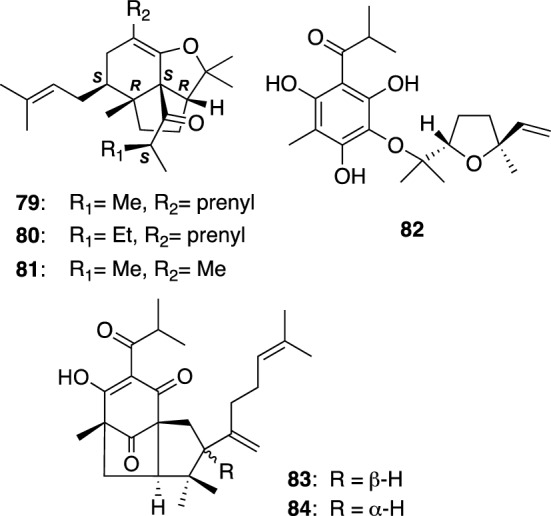
The structures of yezo’otogirins A–F (**79**–**84**) isolated from *Hypericum yezoense*

*Hypericum yezoense* (Yezo-otogiri in Japanese) is a perennial herb grown in the northern area of Japan. The investigation on constituents of the aerial parts of *H. yezoense* collected in Hokkaido gave three PAP-related meroterpenes possessing an unusual fused 6/5/5 tricyclic core, yezo’otogirins A–C (**79**–**81**) (Fig. 10) [51]. We assigned the absolute configurations of **79**–**81** by interpretation of ECD spectra aided with conformational analysis. George et al. achieved the biomimetic total synthesis of (±)-yezo’otogirin A [52]. Furthermore, the total synthesis and a moderate cytotoxicity against human cancer cell lines of (±)-yezo’otogirin C were reported by He and Lee et al. [53, 54]. Yezo’otogirins D–H (**82**–**86**) were isolated from the aerial parts of *H. yezoense* cultivated at Hokkaido [55]. Yezo’otogirins G (**85**) and H (**86**) are simple linear meroterpenes with an enolized *β*-diketone moiety possessing a weak antimicrobial activity against *B. subtilis* and *T. mentagrophytes*, and are structurally related to yojironins A (**77**) and B (**78**) (Fig. 9). Yezo’otogirin D (**82**) is an acylphloroglucinol with a monoterpene moiety linked through an ether bond, while yezo’otogirins E (**83**) and F (**84**) are PAPs possessing a bicyclo[3.2.1]-octane-2,4,8-trione core (Fig. 10). Yezo’otogirin E (**83**) exhibited antimicrobial activites against *Escherichia coli* (MIC 4.0 μg/mL) and *S. aureus* (MIC 8.0 μg/mL) [55].

**Fig. 11:**
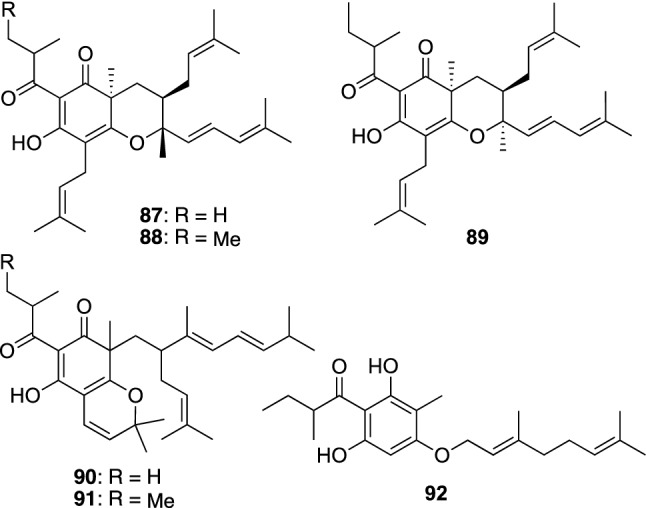
The structures of erecricins A–E (**87**–**91**) and adotogirin (**92**) isolated from *Hypericum erectum*

*Hypericum erecturm* is a perennial herb widely distributed in east Asia. This plant is called “Otogirisou” in Japanese and a representative species of *Hypericum* plants seen in Japan. The aerial parts of *H. erectum* have been used as a traditional remedy to heal wounds, burn wounds, bruises, swelling, and rheumatism. Interestingly, the aerial parts of *H. erectum* were also used for treating disorders of birds. We, however, had an interest in the root constituents of *H. erectum*, and investigated them to isolated PAPs named erecricins A–E (**87**–**91**) and adotogirin (**92**) (Fig. 11) [56]. Erecricins A–E (**87**–**91**) are PAPs possessing a chromane or a chromene skeleton. Adotogirin (**92**), a simple acylphloroglucinol with an *O*-geranyl moiety, displayed antimicrobial activities against MRSA {MIC range 0.5–4.0 μg/mL for seven strains (MIC_50_ 1.0 μg/mL)}, methicillin-sensitive *Staphylococcus aureus* (MSSA) (MICs 1.0 μg/mL for five strains), and *B. subtilis* (MIC 2.0 μg/mL), while **87**–**91** did not show any antimicrobial activities [56].

## Ketides from *Hypericum frondosum* ‘Sunburst’ (section *Myriandra*)

**Fig. 12:**
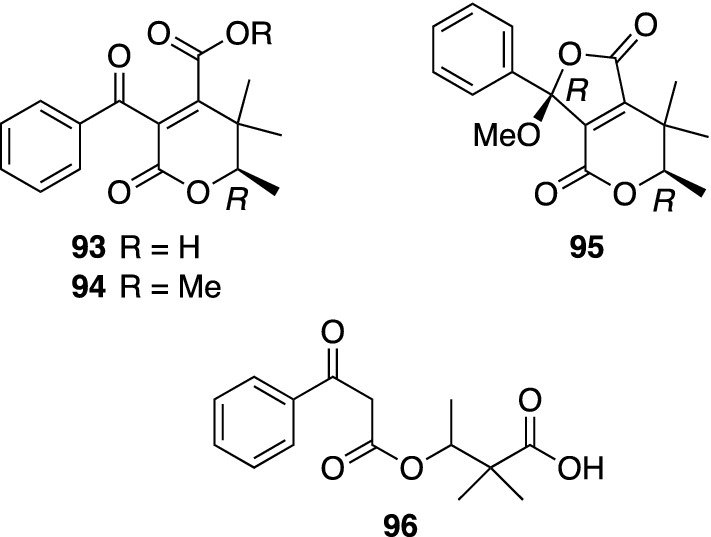
The structures of frondhyperins A–D (**93**–**96**) isolated from *Hypericum frondosum* ‘Sunburst’

Some woody *Hypericum* plants are cultivated as ornamental plants because of their beautiful yellow flowers that bloom in early summer. *H. frondosum* ‘Sunburst’ is a cultivar with larger flowers, and the investigation on the aerial parts of this plant cultivated at the botanical garden of Tokushima University gave four new ketides, frondhyperins A–D (**93**–**96**) (Fig. 12) [57]. Frondhyperins A–D (**93**–**96**) had novel chemical structures comprising short ketide and phenylketide moieties in common. The absolute configuration of **94** was assigned by ECD calculation, while those of **93** and **95** were revealed by their chemical correlation of **94**. Frondhyperin D (**96**) was shown to be a racemate. It is noteworthy that PAPs, common constituents of *Hypericum* plants, were not found in this plant material in our study, although frondhyperin B (**94**) was isolated as a major constituent (325 mg from 870 g of dried aerial parts) [57].

## PAPs from *Hypericum elodeoides* (section Elodeoida) and *H. scabrum* (section Hirtella)

**Fig. 13:**
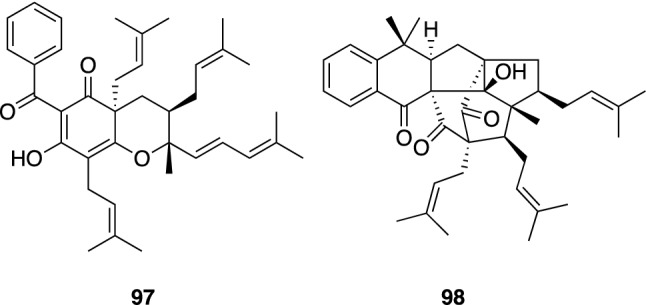
The structures of hypelodins A (**97**) and B (**98**) isolated from *Hypericum elodeoides*

*Hypericum elodeoides* and *H. scabrum* are medicinally used perennial herbs grown in central to west regions of China and in central Asia, respectively. *H. elodeoides* has been used for the treatment of diarrhea and snake bite in China. Chromatographic separations of the extract from the aerial parts of *H. elodeoides* collected in Yunnan province, China furnished two PAPs, hypelodins A (**97**) and B (**98**) (Fig. 13) [58]. Hypelodin A (**97**) is a bicyclic PAP with three prenyl groups and one 4-methyl-1,3-pentadiene moiety, while hypelodin B (**98**) has a cage-like structure with a 6/6/5/7/6/5 hexacyclic ring system. Recently, Park et al. isolated hyperlodin B (**98**) from *H. ascyron* and reported its inhibitory activity against human neutrophil elastase [59].

**Fig. 14:**
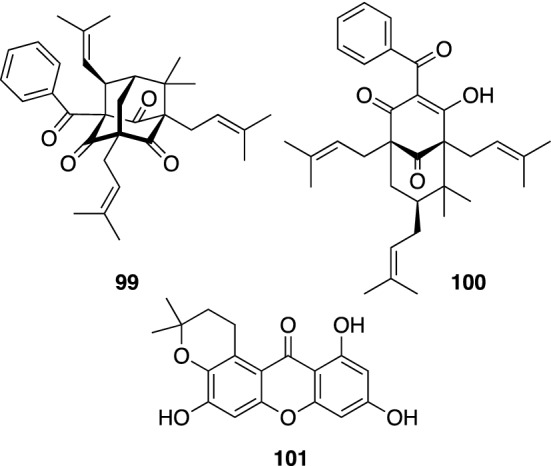
The structures of hyperibones K (**99**) and L (**100**) and hyperxanthone E (**101**) isolated from *Hypericum scabrum*

*H. scabrum* is one of the most popular medicinal herbs in Uzbekistan to treat numerous disorders, such as liver, gall bladder, intestinal, and heart diseases, rheumatism, and cystitis. Investigation on constituents of the aerial parts of *H. scabrum* collected at Chimgan, Uzbekistan showed this plant to be a rich source of polycyclic PAPs with a benzoyl group as their acyl moieties. Hyperibone K (**99**) is the first example of type B PAP possessing a “diamond-like” adamantane skeleton (Fig. 14) [60], whereas a number of type A adamantane or homoadamantane polycyclic PAPs have been reported to date [4, 5]. The absolute configuration of hyperibone K (**99**) was assigned based on the enantioselective total synthesis of an enantiomer of **99** by Porco, Jr. et al. [61]. Hyperibone L (**100**) is a polycyclic PAP with bicyclo[3.3.1]nonane-2,4,9-trione core (Fig. 14) [60]. The synthesis of hyperibone L (**100**) was also achieved by Plietker et al. [62]. We reported a moderate cytotoxicity of hyperibones K (**99**) and L (**100**) against human cancer cell lines (A549 and MCF-7) [60], while a neuroprotective effect on the glutamate-induced toxicity in SK-N-SH cells and a hepatoprotective activity against paracetamol-induced HepG2 cell damage of **99** were reported by Gu et al. [63]. We also isolated prenylated xanthones, hyperxanthones A–F [60], from the same plant material. An inhibitory effect of hyperxanthone E (**101**) (Fig. 14) on interferon-γ plus LPS-induced NO production in RAW 264.7 cells was reported by Xu et al. [64].

## PAPs from *Triadenum japonicum*

**Fig. 15:**
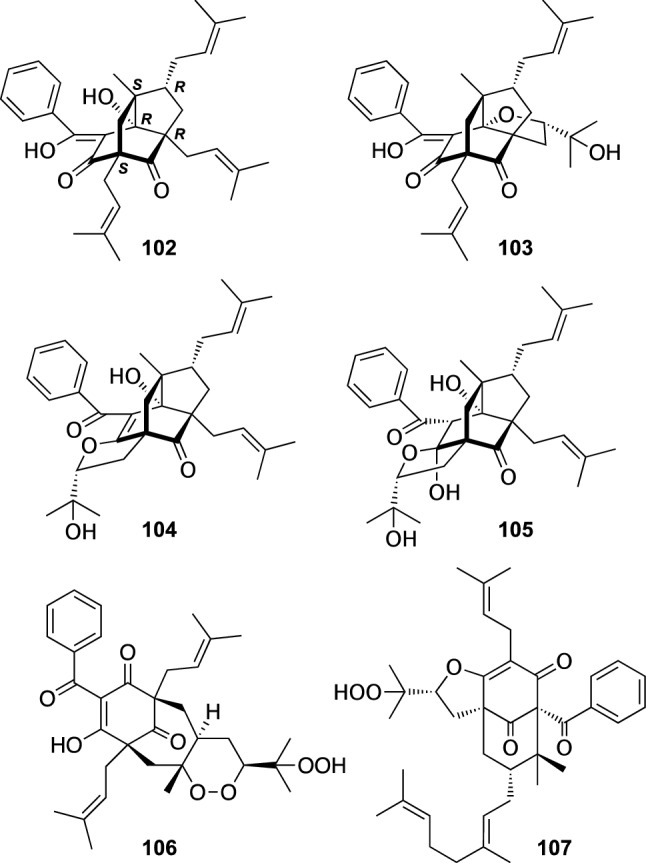
The structures of (−)-nemorosonol (**102**) and trijapins A–E (**103**–**107**) isolated from *Triadenum japonicum*

*Triadenum* is a sister genus of *Hypericum* consisting of six species. *T. japonicum*, a perennial herb bearing small pale pink flowers in contrast with yellow flowers of *Hypericum* plants, grows in marshy places in the eastern Asia and coastal area of eastern Russia. Our phytochemical investigation on the aerial parts of *T. japonicum* collected at Hokkaido resulted in the isolation of six new PAPs, (−)-nemorosonol (**102**) and trijapins A–E (**103**–**107**) [65]. The structure including the absolute configuration of **102** was assigned by NMR analysis and TDDFT ECD calculation. Interestingly, **102** was an enantiomer of (+)-nemorosonol previously isolated from *Clusia nemorosa* (Clusiaceae) [66]. Trijapins A–C (**103**–**105**) were assigned as analogues of (−)-nemorosonol (**102**) with an additional tetrahydrofuran ring, whereas trijapin D (**100**) was shown to be a PAP with an endperoxy moiety. (−)-Nemorosonol (**102**) exhibited antimicrobial activities against *A. niger* (IC_50_ 16 μg/mL), *T. mentagrophytes* (IC_50_ 8 μg/mL), *C. albicans* (IC_50_ 32 μg/mL), *E. coli* (MIC 8 μg/mL), *S. aureus* (MIC 16 μg/mL), *B. subtilis* (MIC 16 μg/mL), and *M. luteus* (MIC 32 μg/mL), while trijapin D (**106**) showed an antimicrobial activity against *C. albicans* (IC_50_ 8 μg/mL) [65] (Fig. 15).

## Conclusion

This review summarized the chemical structures of 107 characteristic metabolites isolated from 11 *Hypericum* plants and one *Triadenum* plant by our research. Their structures were elucidated mainly on the basis of NMR, MS, X-ray, and ECD analyses including a TDDFT ECD calculation method, which has been widely applied to assignment of the absolute configuration of natural products in recent years [67]. Interesting biological activities of the characteristic metabolites, such as antiviral activities against HIV and HCV, antiproliferative activities against cancer cell lines including MDR cancer cell lines, and antimicrobial activities against various bacteria and fungus were also demonstrated. Our phytochemical studies suggested that *Hypericum* plants are a rich source of not only well-known PAPs and xanthones but also meroterpenes. Biyoulactones A–E (**24**–**28**) isolated from *H. monogynum*, hypatulins A (**44**) and B (**45**) isolated from *H. patulum*, and yezo’otogirins A–C (**79**–**81**) isolated from *H. yezoense* were meroterpenes structurally and biosynthetically related to PAPs, while plausible biosynthetic pathway of the PAPs was summarized in previous reviews [4, 5]. In contrast, some meroterpenes were conjugates with unprecedented structures composed of sesquiterpenes and a dibenzo-1,4-dioxane derivative {hyperdioxane A (**18**) isolated from *H. ascyron*} or a spirolactone derivative {biyouyanagins A (**29**) and B (**30**) isolated from *H. monogynum*}. Simple meroterpenes {yojironins A (**77**) and B (**78**) isolated from *H. yojiroanum* and yezo’otogirins D (**85**) and E (**86**) isolated from *H. yezoense*} and ketides {frondhyperins A–D (**93**–**96**) isolated from a cultivar *H. frondosum* ‘Sunburst’} were also biogenetically interesting compounds. Thus, *Hypericum* plants are an attractive source of various characteristic metabolites, and therefore a systematic biological evaluation of our compounds isolated from *Hypericum* plants is in progress.
